# Open access, a revolution in scholarly publishing

**DOI:** 10.3402/jchimp.v1i1.6350

**Published:** 2011-05-09

**Authors:** David J Solomon

**Affiliations:** The Department of Medicine and OMERAD, Michigan State University, USA

I would like to thank Robert Ferguson, MD and the Editorial Team of the *Journal of Community Hospital Internal Medicine Perspectives* (JCHIMP) for the opportunity to write this commentary and congratulate them on the launch of the Journal which will serve as a valuable resource for physicians and other health professionals practicing in community settings. I am also very appreciative of their decision to publish JCHIMP as an open access (OA) journal which will add significantly to its value and impact.

Open access journals are defined by Peter Suber as freely available online and generally free of most copyright and licensing restrictions (1). Since there is a significant cost to distributing paper copies of journals, the only viable means of funding traditional journals has been by charging for each copy, usually through subscription fees. With digital publication there are essentially no incremental costs for distribution. While publishing a quality scholarly journal such as JCHIMP requires considerable resources, the resources are the same no matter how many copies are distributed. This allows the possibility of distributing the journal at no charge and seeking other means for funding the cost of publication.

Why would we want to move away from the subscription model of funding journals even when there is no cost for distribution? The answer can be summed up in one word, access. Peer reviewed scholarly journals have served for several hundred years as our mainstay of scientific communication and have formed the world's most up-to-date and authoritative archive of knowledge. Medical research as well as innovations in how we deliver health care are dependent on the free flow of information though journals such as JCHIMP. The more accessible these journals are to all who need them, the more quickly medical practice will evolve, and the better able we will be able to apply the knowledge gained in providing patient care.

OA streamlines access to peer-reviewed journals. Accessing an OA article identified through a PubMed search takes one click on a hyperlink. For a subscription journal you will instead be taken to the publisher's web site and an invitation to spend around $30.00 to access the article. It is true that most physicians and other health professionals have reasonably good access to the medical literature through their institutional libraries, but accessing the literature is generally not nearly as simple as ‘clicking’ on a hyperlink from where it is referenced. It generally means going to the library's web portal, then linking to the publisher's web site, and drilling down to the journal, volume, issue, and finally the desired article. In my experience this takes several tedious minutes. Although this seems like a small price to pay, the time and effort adds up and serves no real purpose other than to support an inefficient and cumbersome system for funding publication. Furthermore, no library collection is complete. Despite working at a Big 10 University, I often find our library does not have a subscription to the journal articles I need and are forced to seek them through an inter-library loan, which is even more cumbersome and often takes several weeks.

We are also the lucky ones. Libraries in the developing world generally lack the funds for even the most basic set of subscription journals. Although the World Health Organization's HINARI program (2) is a major step forward, it has not solved the problem of accessing the literature in the developing world. When it costs nothing extra to provide practitioners throughout the world the latest knowledge in medicine and public health, it seems unethical not to make it available.

Patients are increasingly turning to the Web for information about their health problems. Sometimes they obtain the information from quality sources such as Medline Plus and sometimes not. People with chronic illnesses and their family members often wish to obtain the most up-to-date information possible on their condition and are willing to invest the time and effort necessary to read and understand the primary literature. Well-educated patients and their families are better able to partner with their physicians in determining the best course of treatment and are more likely to comply with their recommendations with well-documented improvements in health outcomes (3, 4). Unfortunately without easy access to medical libraries they are often forced to pay an exorbitant price to access research results that their own tax dollars have funded.

Devising a systematic means for funding OA remains the most daunting challenge to implementing the model. On that note we all owe a great deal of gratitude to Union Memorial Hospital and Medstar Health for generously providing much of the funding for making JCHIMP a reality. Unfortunately most OA journals do not have such generous benefactors and there is no guarantee these organizations will be able to fund JCHIMP indefinitely. The OA publication does not cost any more than subscription publication and most of the funding for subscription publication comes from a variety of public sources funneled through our university libraries. We need to reorganize the funding streams that have evolved over decades to support a subscription publication model. This fortunately is beginning to happen both through governmental regulations (5) and by university initiatives (6), though not as quickly as those of us who advocate for OA might like. We have made considerable progress and the medical literature is increasingly becoming freely available. A recent study estimated that in 2009, 13.9% of the medical literature was available through OA journals and another 7.8% was available through authors placing articles from subscription journals in public archives like PubMed Central (7). I am pleased that JCHIMP will add to this growing trend.
